# Magnetic Targeted Delivery of Induced Pluripotent Stem Cells Promotes Articular Cartilage Repair

**DOI:** 10.1155/2017/9514719

**Published:** 2017-12-26

**Authors:** Shinji Kotaka, Shigeyuki Wakitani, Akira Shimamoto, Naosuke Kamei, Mikiya Sawa, Nobuo Adachi, Mituo Ochi

**Affiliations:** ^1^Department of Orthopaedic Surgery, Division of Medicine, Biomedical Sciences Major, Graduate School of Biomedical Sciences, Hiroshima University, Kasumi 1-2-3 Minami-ku, Hiroshima 734-8551, Japan; ^2^Graduate School of Health and Sports Sciences, Mukogawa Women's University, 6-46 Ikebiraki, Nishinomiya, Hyogo 663-8558, Japan; ^3^Department of Cellular and Molecular Biology, Institute of Biomedical & Health Sciences, Hiroshima University, Hiroshima 734-8551, Japan

## Abstract

Cartilage regeneration treatments using stem cells are associated with problems due to the cell source and the difficulty of delivering the cells to the cartilage defect. We consider labeled induced pluripotent stem (iPS) cells to be an ideal source of cells for tissue regeneration, and if iPS cells could be delivered only into cartilage defects, it would be possible to repair articular cartilage. Consequently, we investigated the effect of magnetically labeled iPS (m-iPS) cells delivered into an osteochondral defect by magnetic field on the repair of articular cartilage. iPS cells were labeled magnetically and assessed for maintenance of pluripotency by their ability to form embryoid bodies *in vitro* and to form teratomas when injected subcutaneously into nude rats. These cells were delivered specifically into cartilage defects in nude rats using a magnetic field. The samples were graded according to the histologic grading score for cartilage regeneration. m-iPS cells differentiated into three embryonic germ layers and formed teratomas in the subcutaneous tissue. The histologic grading score was significantly better in the treatment group compared to the control group. m-iPS cells maintained pluripotency, and the magnetic delivery system proved useful and safe for cartilage repair using iPS cells.

## 1. Introduction

Articular cartilage is known for its poor regenerative and reparative ability, making repair difficult after injury due to insults such as trauma, osteoarthritis, or rheumatoid arthritis. Current treatments for cartilage injury include conservative treatments such as rehabilitation, anti-inflammatory analgesic medication, and intra-articular injection or operative treatments such as bone marrow stimulating techniques (drilling and microfracture) and autologous osteochondral grafting [1, 2]. However, there are problems associated with these methods. Bone marrow stimulating techniques and autologous osteochondral grafting are unable to completely restore hyaline cartilage. Cartilage regeneration is one of the prime targets that remains largely unsolved [1, 3].

Recently, there have been many reports of cartilage regeneration treatment using stem cells. Recently reported studies on cartilage regeneration have used MSCs, as well as stem cells derived from adipose tissue, synovial tissue, and peripheral blood [4–6]. Vega and collaborators reported significantly better function and cartilage quality in osteoarthritis patients treated with MSCs by intra-articular injection [7]. However, major disadvantages of MSCs include limitations *in vitro* proliferative potential, and their proliferative capacity and synthetic capacity decline with age [8].

Embryonic stem (ES) cells and induced pluripotent stem (iPS) cells are thought to be an ideal cell source for tissue regeneration. We reported that ES cells can be differentiated into cartilage and used to repair defects when placed in a cartilage defect [9]. However, the use of these cells raises ethical issues since ES cells are derived from fertilized human eggs. On the other hand, there are no ethical issues associated with the use of iPS cells because they are induced from mature somatic cells, and a large number of cells can easily be collected. A paper by Ko et al. reported the use of human iPS cells implanted into cartilage defects and showed that the defect was filled with good quality cartilage [10].

We reported that when ES cells were transplanted into the knee joint, they formed tumors and destroyed the knee joint in SCID mice [11]. However, when they were transplanted into an osteochondral defect, they did not generate teratomas. These results demonstrate that it is important to confine the ES cells to the defect. It is conceivable that some growth factors are released from bone marrow which promote the chondrogenesis of ES cells [9]. On the other hand, Kamei et al. reported delivery of magnetically labeled mesenchymal stem cells into an osteochondral defect using a magnetic field, resulting in good repair of the defects [12]. Consequently, we hypothesized that if magnetically labeled iPS cells could be delivered specifically into cartilage defects by magnetic field, it would be possible to prevent the formation of teratomas and to repair articular cartilage. The purpose of this study was to investigate the efficacy and safety of magnetic targeting of iPS cells for articular cartilage repair.

## 2. Materials and Methods

### 2.1. iPS Cell Preparation

Human iPS cells, derived from human fetal lung cells (MRC-5) and infected with recombinant retroviruses expressing the four reprogramming factors (Oct3/4, Sox2, Klf4, and c-Myc), were purchased from the National Institutes of Biomedical Innovation, Health and Nutrition. The cell number is JCRB1331 [13].

Feeder cells were prepared from mouse primary embryonic fibroblasts (MEF) inactivated with mitomycin C. The iPS cells were cultured on the feeder cells. The medium (Serum-free Essential 8 Medium; Life Technologies, California, USA) was changed every day.

### 2.2. Animals

Nine- to ten-week-old nude rats (F344/NJcl-rnu/rnu) used in this study were purchased from CLEA Japan Inc. (Tokyo, Japan). This study was approved by the Committee of Research Facilities for Laboratory Animal Science (Graduate School of Biomedical Science, Hiroshima University), and rats were cared for according to the Guide for Animal Experimentation.

### 2.3. External Magnetic Force

To deliver a magnetic field, we used a neodymium magnet (Sangyo Supply Inc., Miyagi, Japan).

### 2.4. Magnetic Labeling of iPS Cells

Serum-free Essential 8 medium with 15% fetal bovine serum (FBS) and 1% antibiotic mixed stock solution (Nacalai Tesque Inc., Kyoto, Japan) were equilibrated at 37°C under 5% CO_2_ for at least 30 minutes. Nanoscale iron particles (ferucarbotran; 27.9 mg Fe/mL) (Fujifilm RI Pharma Co. Ltd., Tokyo, Japan) were added to the solution at a concentration of 98.2 mg Fe/mL. The solution was agitated for 3 to 5 minutes by manual shaking, then added to the iPS cells in the culture dish, and cultured overnight. On the next day, the dish was washed twice with sterile phosphate-buffered saline (PBS).

To purify magnetically labeled iPS cells, an external magnet was placed beside the tube as the nonadherent magnetically labeled iPS cells were dropped into the culture medium. The cells were separated into those attracted towards the magnet and those that fell to the bottom. The iPS cells drawn towards the magnet were denoted as m-iPS cells, while the others were denoted as non-m-iPS cells. The cell number in each group was counted.

### 2.5. Assessment of m-iPS Cells

#### 2.5.1. Evaluating the Capture of Nanoscale Iron Particles into iPS Cells

After magnetic labeling, culture medium was removed and the dish was washed twice with PBS. The cells were fixed in 4% paraformaldehyde phosphate-buffered solution (Wako Pure Chemical Industries Ltd., Osaka, Japan) for 10 minutes at room temperature. The phosphate-buffered paraformaldehyde solution was removed, and the dish was washed with PBS, then dehydrated with 100% ethanol for 10 minutes, and dried in air after removing the ethanol. The dish was stained with Berlin blue to show the presence of iron.

#### 2.5.2. Assessment of Pluripotency

*(1) In Vitro Assessment*. Embryoid bodies were formed as follows. The m-iPS cells were singly dissociated and mixed in primate ES cell medium (ReproCELL Inc., Kanagawa, Japan) with 10 *μ*M Rho-associated protein kinase inhibitor 4-[(1R)-1-aminoethyl]-N-4-pyridinyl-trans-cyclohexanecarboxamide, dihydrochloride (Y-27632; Wako Pure Chemicals, Osaka, Japan). The m-iPS cells were then seeded onto 96-well plates at 1 × 10^4^ cells/well and centrifuged for 3 minutes. After incubating for 3 weeks under a humidified atmosphere of 5% CO_2_ at 37°C, the formed pellets were cultured for 2 weeks, with medium changes every two days. The pellets were then immunostained as follows: following fixation of the pellets with 4% paraformaldehyde phosphate-buffered solution for 15 min at 4°C, cells were permeabilized with 0.1% Triton X-100, washed with PBS containing 2% BSA, and incubated with primary antibodies diluted in PBS containing 2% BSA. Primary antibodies against *β*III-tubulin (Merck Millipore, Darmstadt, Germany), desmin (Thermo Fisher Scientific Inc., Waltham, MA, USA), and *α*-fetoprotein (Merck Millipore) were detected using the secondary antibodies Alexa-fluor® 488-conjugated anti-mouse IgG and Alexa-fluor 488-conjugated anti-rabbit IgG (both from Thermo Fisher Scientific Inc.). Cell nuclei were stained with 1 *μ*g/mL 4′,6-diamidino-2-phenylindole (DAPI) (Thermo Fisher Scientific Inc.).

*(2) In Vivo Assessment*. Teratoma formation was evaluated as follows. Nude rats were used as implant recipients in this study. Aliquots of 1 × 10^8^ m-iPS cells were percutaneously implanted into the subcutaneous area. Once a tumor had formed, the rats were humanely killed, and the tumor was resected to assess tumor formation histologically. Tumors were fixed in 4% paraformaldehyde phosphate-buffered solution for 48 hours and then embedded in paraffin blocks. The samples were cut into 5 *μ*m sections and stained with hematoxylin and eosin for histological assessment.

### 2.6. Surgery

To prepare for transplantation, a single cell suspension of m-iPS cells was prepared and mixed in 3% atelocollagen (Koken, Tokyo, Japan) at 10^7^ cells/mL. Nude rats were used in this study. Before surgery, the rats were given sufficient anesthesia by ketamine (100 mg/kg) and xylazine (12 mg/kg) by intraperitoneal administration. The medial parapatellar approach was used to expose the knee joint, and a defect was created in the patellar groove using a 2 mm steel round burr in both knees. The defect size was 2 mm in both width and depth. The rats were randomly divided into three groups. In the magnetic force group (M group; *n* = 18), the knee defects were filled with m-iPS cells (10^5^ cells) which were drawn into the cartilage defect using an external magnetic force for 10 minutes. The magnet was located on popliteal fossa for drawing into defect. In the nonmagnetic force group (NM group; *n* = 18), the knee defects were filled with iPS cells (10^5^ cells) without the use of an external magnetic force for 10 minutes. For the control group (C group; *n* = 18), the defects were filled with atelocollagen alone. At the completion of the surgery, the patella was repositioned, and the wound was closed. Six rats from each group were humanely killed at each of the time points of 4, 6, and 8 weeks after surgery.

### 2.7. Histological Analyses

The distal femurs were excised and fixed in 4% paraformaldehyde phosphate-buffered solution for 48 hours. The samples were decalcified with ethylenediaminetetraacetic acid (EDTA) solution and embedded in paraffin blocks, which were cut into 5 *μ*m sections along the axial plane, deparaffinized, and hydrated. For histological assessment, the sections were stained with hematoxylin and eosin and with safranin O. For immunohistological assessment, the sections were stained with antibodies against type II collagen and human mitochondria. For immunohistochemical staining with antibodies against type II collagen and human mitochondria, the sections were incubated with 10% immunoactive (Matsunami Glass Ind. Ltd., Osaka, Japan) in PBS. The slides were blocked in hydrogen peroxide solution (3% H_2_O_2_) for 10 minutes, followed by washing with PBS. Primary antibodies were applied and incubated overnight at 4°C. The antibody reaction procedures were followed by a treatment with an avidin-conjugated peroxidase (Vectastain ABC-Elite Lit, Vector Laboratories, Burlingame, California, USA), and then visualization was performed using a 3,3′-diaminobenzidine (DAB) substrate kit (Vector Laboratories). Each sample stained with antibodies against type II collagen was graded according to the histologic grading score for cartilage regeneration as described by us and Pineda et al.

### 2.8. Statistical Analysis

All results are expressed as mean ± standard deviation. Statistical comparisons among multiple groups were evaluated by the Kruskal-Wallis test, and a pairwise comparison was performed using the Mann–Whitney test. A significant difference was set at *P* < 0.05.

## 3. Results

### 3.1. Magnetic Labeling of iPS Cells

**Figure 1:**
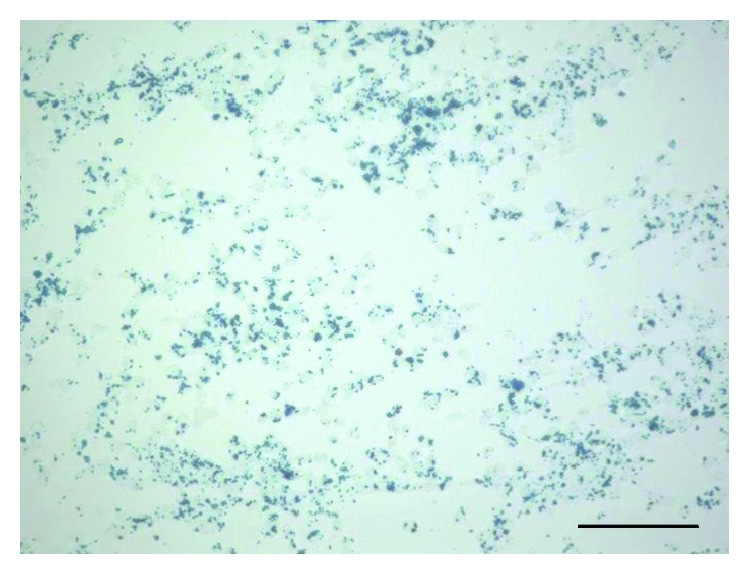
Microscopic findings (Berlin blue stain) of iPS cells labeled magnetically. Nanoscale iron particles were located inside m-iPS cells. Scale bar = 200 *μ*m.

Staining showed that most iPS cells had become magnetically labeled m-iPS cells because nanoscale iron particles were located inside almost all the iPS cells (Figure 1). Cell counts revealed that m-iPS cells made up 93.1 ± 2.2% of the population while non-m-iPS cells made up 6.9 ± 2.2% (*P* < 0001).

### 3.2. Assessment of Pluripotency

#### 3.2.1. *In Vitro* Assessment

**Figure 2:**
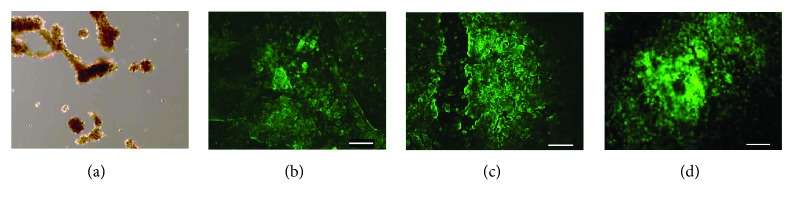
Microscopic findings of m-iPS cell pellets (a). The pellets was evaluated by immunofluorescent stain (b, c, and d). Ectoderm maker alpha-fetoprotein (b), mesoderm maker desmin (c), and endoderm maker beta III-tubulin (d) were expressed from the pellets. So we succeeded in forming embryoid body derived from the m-iPS cells. Scale bar = 500.

Immunofluorescence staining showed that the ectoderm marker alpha-fetoprotein (Figure 2(b)), the mesoderm marker desmin (Figure 2(c)), and the endoderm marker beta III-tubulin (Figure 2(d)) were expressed in the pellets. This confirms that embryoid bodies were successfully derived from the m-iPS cells. These findings suggest that they have the ability to differentiate into three embryonic germ layers and that m-iPS cells maintain pluripotency.

#### 3.2.2. *In Vitro* Assessment

**Figure 3:**
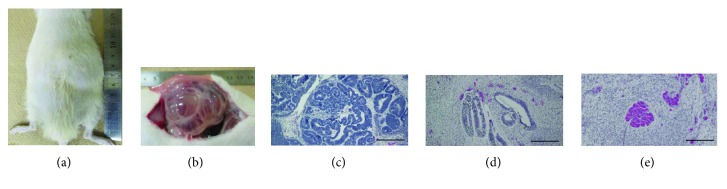
Tumor formation in nude rat in which m-iPS cells were transplanted (a and b). The nude rat in which m-iPS cells were transplanted showed tumor formation at 7 weeks after transplantation (a). Macroscopically, the tumor was solid and cystic and pressed the surrounding tissue outwards (b). Histopathological findings (hematoxylin and eosin stain) of the tumor (c, d, and e). The tumor contains immature neural tube (c), immature glandular structure (d), and immature muscle (e). We conclude that the tumor is immature teratoma. Scale bar = 300 *μ*m.

Seven rats were percutaneously implanted with m-iPS cells into subcutaneous tissue, and two rats showed tumor formation 7 weeks after implantation. Macroscopically, each tumor was a mixture of cystic and solid tissue (Figures 3(a) and 3(b)). Histologically, the tumor was categorized as an immature teratoma, because it contained neural tube (Figure 3(c)), glandular structure (Figure 3(d)), and muscle (Figure 3(e)).

### 3.3. Assessment of Articular Cartilage Repair

**Figure 4:**
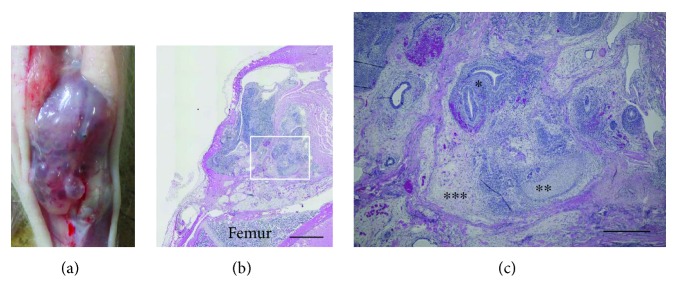
Tumor formation in the knee joints and subcutaneous spaces in the NM group at 4 weeks after transplantation. Macroscopically, the tumor was solid and cystic (a). Histopathological findings of the tumor (hematoxylin and eosin stain, b and c). The tumor contains immature neuroepithelium (^∗^), immature cartilage (^∗∗^), and immature mesenchymal elements (^∗∗∗^). We conclude that the tumor is immature teratoma. Scale bar = 2 mm in (b) and 500 *μ*m in (c).

After transplanting iPS cells embedded in collagen hydrogel into the osteochondral defect of rats, no tumors were seen in the M group. Macroscopic examination of a rat in the NM group showed tumor formation in the knee joint and subcutaneous space at 4 weeks after transplantation (Figure 4(a)). Histologically, the tumor extruded from the knee joint and was thought to be a teratoma (Figure 4(b)).

**Figure 5:**
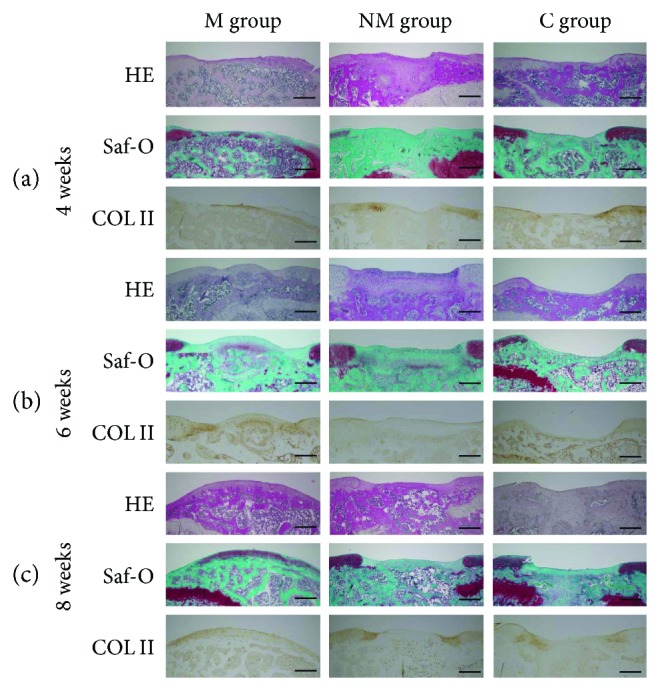
In vivo cartilage repair at 4, 6, and 8 weeks after transplantation. At 4 weeks after transplantation in the M group, osteochondral defects in the patellar groove of the femur were covered by hyaline-like cartilage; however, the surface of cartilage was irregularity (a). At 6 and 8 weeks, defects were also covered by hyaline-like cartilage, and the surface was smooth (b and c). On the other hand, in the NM group and C group, osteochondral defects could be not regenerated cartilage by 8 weeks (c). Scale bar = 500 *μ*m.

**Figure 6:**
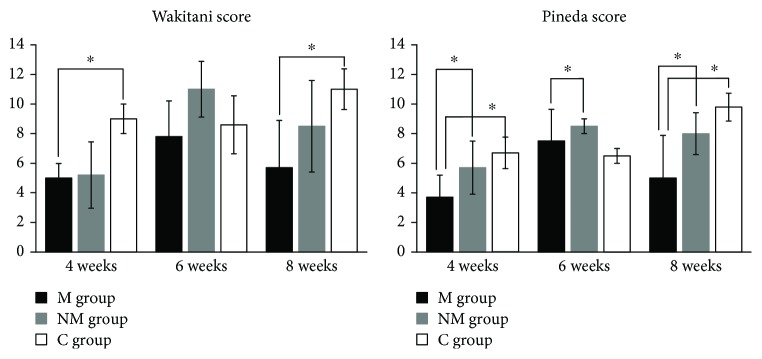
The mean histologic grading score by Wakitani and Pineda. The mean histologic grading score by Wakitani was significantly better in the M group than in the C group (in 4 weeks: *P* = 0.011; in 8 weeks: *P* = 0.005). By Pineda, score was significantly better in the M group than in the other groups (M and NM, in 4 weeks: *P* = 0.05; M and C, in 4 weeks: *P* = 0.002; M and NM, in 8 weeks: *P* = 0.04; M and C, in 8 weeks: *P* = 0.008). ^∗^*P* < 0.05.

**Figure 7:**
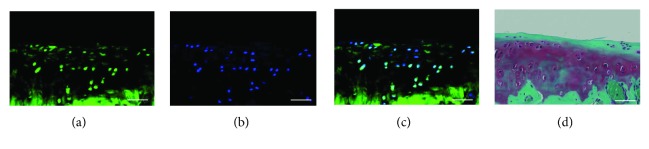
Immunofluorescent staining with antibodies against human mitochondria (a, b, and c) and safranin O staining (d) of cartilage cells. The antibodies against human mitochondria were expressed from cartilage cells. These findings suggest that m-iPS cells differentiate into cartilage cells. Bar = 50 *μ*m.

At 4 weeks after transplantation in the M group, osteochondral defects in the patellar groove of the femur were covered by hyaline-like cartilage; however, the surface of the cartilage was irregular (Figure 5(a)). At 6 and 8 weeks, all defects were covered by hyaline-like cartilage, and the surface was smooth (Figures 5(b) and 5(c)). In contrast, in the NM and C groups, osteochondral defects showed no regenerated cartilage by 8 weeks (Figure 5(c)). In addition, the mean histologic grading score, according to the method of Wakitani, was significantly better in the M group than in the C group (at 4 weeks: *P* = 0.011; at 8 weeks: *P* = 0.005) (Figure 6(a)). Using the method of Pineda, the score was significantly better in the M group than in the other groups (M and NM, at 4 weeks: *P* = 0.05; M and C, at 4 weeks: *P* = 0.002; M and NM, at 8 weeks: *P* = 0.04; M and C, at 8 weeks: *P* = 0.008) (Figure 6(b)). In the M group, immunofluorescent staining showed that human mitochondria were present in the cartilage cells. These findings suggest that m-iPS cells differentiate into cartilage cells (Figure 7).

## 4. Discussion

In this study, we found that the repair of cartilage defects using m-iPS cells with application of an external magnetic force was significantly better than without the external magnetic force. Furthermore, we found no evidence of tumor formation in the knee joint transplanted with m-iPS cells with external magnetic force, while those transplanted without external magnetic force did show tumor formation. The results demonstrated that this system for delivery of iPS cells makes cartilage regeneration possible and prevents teratoma formation.

We have shown that iPS cells can be magnetically labeled with nanoscale iron particles and still maintain pluripotency, demonstrated by the formation of embryoid bodies and teratomas from m-iPS cells. There have been several previous reports of iPS cells labeled magnetically. Castaneda and collaborators reported that iPS cells were labeled magnetically using ferumoxytol and visualized with MR imaging [14]. Furthermore, Ruan and collaborators reported that embryoid bodies were formed from iPS cells labeled magnetically and that these cells maintained pluripotency and differentiated into three germ layers *in vitro* [15].

However, there has been no report of cartilage regeneration using iPS cells labeled magnetically. This study is the first to evaluate the repair of a cartilage defect using iPS cells labeled magnetically in combination with an external magnetic force. m-iPS cells were delivered to the cartilage defect of the knee joint by an external magnetic field, and the defect was covered by hyaline-like cartilage. Conversely, when m-iPS cells were transplanted into the defect without the use of an external magnetic field, the defect was not covered by hyaline-like cartilage. In addition, the histologic grading score for cartilage regeneration was significantly better in the former than in the latter. Uto and collaborators reported transplanting iPS cells combined with collagen hydrogel into an osteocartilage defect and found that the defects were repaired [3]. They concluded that mixing iPS cells in collagen hydrogel could cause these cells to undergo a loss of cell-cell contact, which forced the iPS cells to lose pluripotency and undergo differentiation. In this study, m-iPS cells embedded in atelocollagen were delivered into a cartilage defect by an external magnetic field and the cartilage showed a good level of repair. However, when m-iPS cells embedded in atelocollagen were transplanted without an external magnetic field, the cartilage repair was not as good. We believe that the m-iPS cells were not drawn toward the defect without the external magnetic field; consequently, fewer cells were present within the defect and the cartilage repair was not as effective.

Yamashita and collaborators reported that differentiation of iPS cells into hyaline cartilaginous particles and implantation of the particles into joint surface defects in immunodeficient rats prevented teratoma formation. As a result, cartilage defects were repaired and neither tumors nor ectopic tissue formation was observed [16]. However, *in vivo* culture expansion of cartilage stem cells is associated with disadvantages including labor, high cost, the necessity for expensive growth factors, and the risk of infection [17, 18]. We reported that when ES cells were injected into the knee joint of SCID mice, the cells formed teratomas. However, when the ES cells were transplanted into osteochondral defects in the patellar groove of immunosuppressed rats treated with cyclosporine, no sign of tumor growth or nonchondrocyte tissue was observed in the transplant recipients and the defects were repaired with hyaline-like cartilage. The reasons may be that cells in the joint space are fed by joint fluid not by blood, while on the other hand, those in an osteochondral defect are fed by both joint fluid and blood. Some growth factors released from bone marrow promote the chondrogenesis of ES cells [9]. In this study, m-iPS cells were delivered to a cartilage defect in the knee joint by an external magnetic field and moved to the bottom of cartilage defect, and no tumor formation was observed. In contrast, when m-iPS cells were transplanted into the defect without the use of an external magnetic field, teratoma formation was observed at seven weeks after transplantation. The tumor appeared to be an immature teratoma, based on histological analysis. We believe that iPS cells differentiated into hyaline-like cartilage or teratoma by the same mechanism as ES cells.

## 5. Conclusions

This study has demonstrated that magnetically labeled iPS cells maintain pluripotency and that when they are delivered to a cartilage defect by an external magnetic field, they can regenerate cartilage. In addition, the histologic grading score in the M group was significantly better than in the NM group or in the C group, and no tumor formation was observed. This method will be useful for cartilage repair using iPS cells.
